# A Novel Clinical-Radiomics Model Based on Sarcopenia and Radiomics for Predicting the Prognosis of Intrahepatic Cholangiocarcinoma After Radical Hepatectomy

**DOI:** 10.3389/fonc.2021.744311

**Published:** 2021-11-19

**Authors:** Liming Deng, Bo Chen, Chenyi Zhan, Haitao Yu, Jiuyi Zheng, Wenming Bao, Tuo Deng, Chongming Zheng, Lijun Wu, Yunjun Yang, Zhengping Yu, Yi Wang, Gang Chen

**Affiliations:** ^1^ Department of Hepatobiliary Surgery, The First Affiliated Hospital of Wenzhou Medical University, Wenzhou, China; ^2^ Key Laboratory of Diagnosis and Treatment of Severe Hepato-Pancreatic Diseases of Zhejiang Province, The First Affiliated Hospital of Wenzhou Medical University, Wenzhou, China; ^3^ Department of Medical Imaging, The First Affiliated Hospital of Wenzhou Medical University, Wenzhou, China; ^4^ Department of Epidemiology and Biostatistics, School of Public Health and Management, Wenzhou Medical University, Wenzhou, China

**Keywords:** sarcopenia, clinical-radiomics model, intrahepatic cholangiocarcinoma, radical hepatectomy, nomogram

## Abstract

**Background:**

Intrahepatic cholangiocarcinoma (ICC) is a highly aggressive malignant tumor with a poor prognosis. This study aimed to establish a novel clinical-radiomics model for predicting the prognosis of ICC after radical hepatectomy.

**Methods:**

A clinical-radiomics model was established for 82 cases of ICC treated with radical hepatectomy in our hospital from May 2011 to December 2020. Radiomics features were extracted from venous-phase and arterial-phase images of computed tomography. Kaplan-Meier survival analysis was generated to compare overall survival (OS) between different groups. The independent factors were identified by univariate and multivariate Cox regression analyses. Nomogram performance was evaluated regarding discrimination, calibration, and clinical utility. C-index and area under the curve (AUC) were utilized to compare the predictive performance between the clinical-radiomics model and conventional staging systems.

**Results:**

The radiomics model included five features. The AUC of the radiomics model was 0.817 in the training cohort, and 0.684 in the validation cohort. The clinical-radiomics model included psoas muscle index, radiomics score, hepatolithiasis, carcinoembryonic antigen, and neutrophil/lymphocyte ratio. The reliable C-index of the model was 0.768, which was higher than that of other models. The AUC of the model for predicting OS at 1, and 3 years was 0.809 and 0.886, which was significantly higher than that of the American Joint Committee on Cancer 8^th^ staging system (0.594 and 0.619), radiomics model (0.743 and 0.770), and tumor differentiation (0.645 and 0.628). After stratification according to the constructed model, the median OS was 59.8 months for low-risk ICC patients and 10.1 months for high-risk patients (*p <* 0.0001).

**Conclusion:**

The clinical-radiomics model integrating sarcopenia, clinical features, and radiomics score was accurate for prognostic prediction for mass-forming ICC patients. It provided an individualized prognostic evaluation in patients with mass-forming ICC and could helped surgeons with clinical decision-making.

## Introduction

Intrahepatic cholangiocarcinoma (ICC) is a malignant tumor, which accounts for 10-15% of primary liver cancer (1). In the past 30 years, the incidence of ICC has been on the rise worldwide (2), and it is 0.97 to 7.55 cases per 100,000 person-years in China (3). ICC can be classified into four types: mass-forming, intraductal growth, periductal infiltration, and mixed growth, among which, mass-forming type accounts for 85% (4). ICC has a poor prognosis due to its insidious onset, high aggressiveness, and lack of effective treatment (5). Radical hepatectomy is the only effective treatment for ICC (6), but it is still easy to relapse and metastasize, and the expected 5-year overall survival (OS) rate of postoperative patients is 25-40% (5–8). ICC patients have a dismal postoperative prognosis; therefore, it is crucial to identify the prognostic factors for ICC that could help surgeons make personalized precision treatment.

Radiomics was first proposed by Lambin (9). It is a technique to quantify tumor heterogeneity by high-throughput extraction of quantitative imaging features such as texture and morphology of lesions from computed tomography (CT), magnetic resonance imaging, and positron emission tomography images, and has become a method to evaluate tumor phenotypes and guide clinical decision-making (10–12). It has been used in the diagnosis, biological behavior prediction, and post-treatment evaluation of ICC (13–17). A radiomics model can accurately predict the clinical outcome of patients with ICC and is a promising prognostic model.

Sarcopenia is an independent disease (18). The European Working Group on Sarcopenia in Older People (EWGSOP) states that people with low muscle strength and mass can be diagnosed with sarcopenia (19). The psoas muscle index (PMI) is a simple and convenient measurement index that can well reflect sarcopenia (20–22). Recently, researchers have paid more attention to the role of sarcopenia in tumors, and several studies have determined that sarcopenia is a significant prognostic factor for patients with ICC (22–24).

Currently, although the OS of ICC patients treated with surgery is still poor, the prognosis of ICC patients varies among individuals owing to tumor heterogeneity (25). Therefore, research of individualized prognostic predictive models has become a research hotspot (26–28). However, studies that combined radiomics with sarcopenia to establish a model have not been reported. Therefore, our study aimed to establish prognostic models for patients who underwent radical surgery for mass-forming ICC, which is based the radiomics, sarcopenia, and clinical features of ICC patients.

## Patients and Methods

### Patients Selection

**Figure 1:**
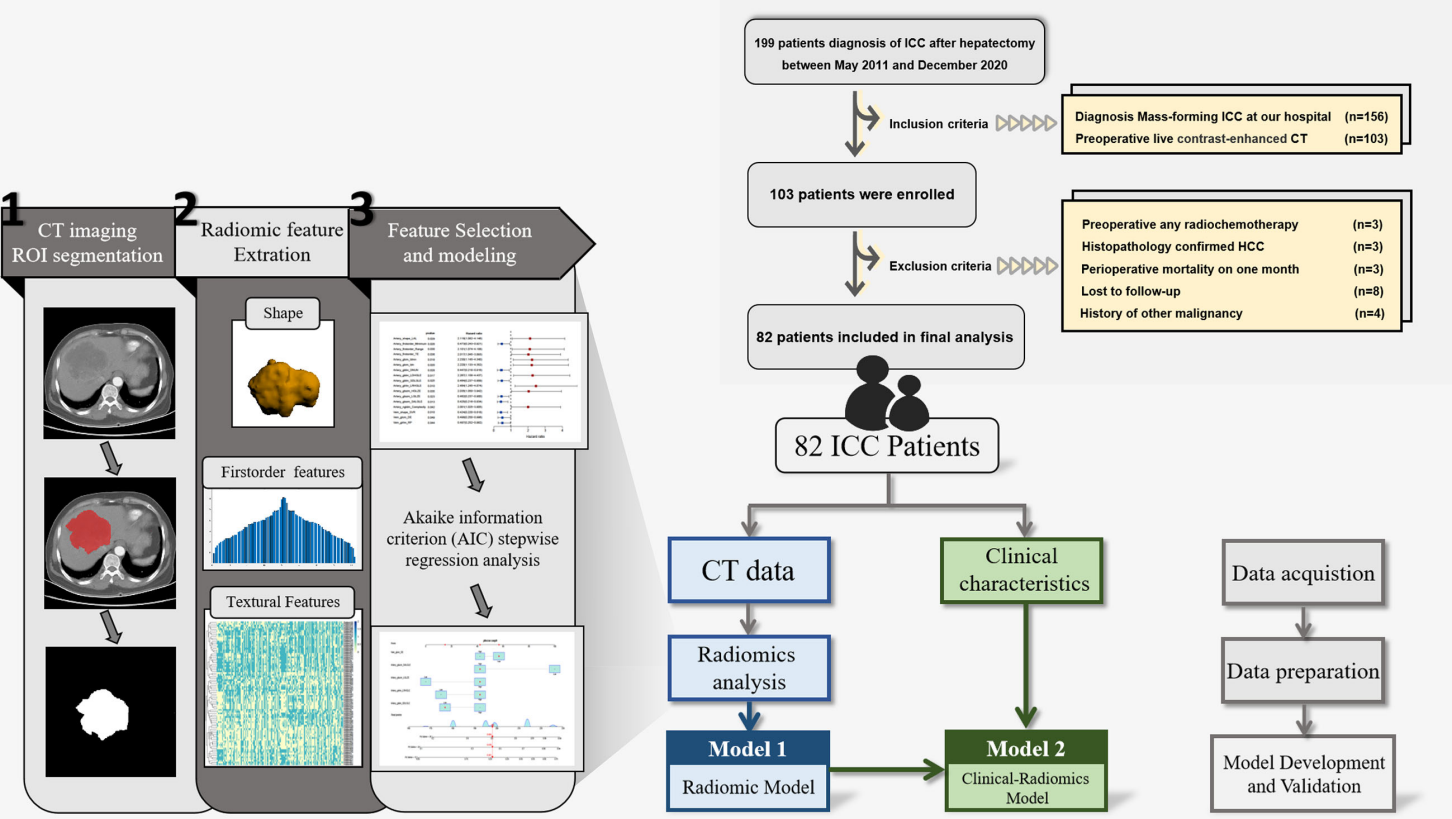
Flow chart of the study.

The present retrospective study was based on data from ICC patients treated with radical hepatectomy at the First Affiliated Hospital of Wenzhou Medical University between May 2011 and December 2020. The patients who met the following conditions were selected to form the final cohort: (1) diagnosed with mass-forming ICC; and (2) contrast-enhanced CT was performed within 1 month before surgery. The exclusion criteria were: (1) patients who received preoperative any radiochemotherapy; (2) patients confirmed with combined ICC and hepatocellular carcinoma; (3) perioperative death; (4) patients lost during follow-up; and (5) history of other malignancy. The patient recruitment and selection criteria are illustrated in **Figure 1**.

All procedures of this retrospective cohort study and the ethical issues involved were reviewed and approved by the Institutional Review Board of the First Affiliated Hospital of Wenzhou Medical University.

### Clinical Variables Collection

Demographic characteristics included age, gender, American Society of Anesthesiologists (ASA) score, and body mass index (BMI). Laboratory variables were taken from the results of a test closest to the date of surgery, included hepatitis B surface antigen (HBsAg), α-fetoprotein (AFP), carcinoembryonic antigen (CEA), carbohydrate antigen 19-9 (CA19-9), albumin (ALB), neutrophil/lymphocyte ratio (NLR), platelet/lymphocyte ratio (PLR), and lymphocyte/monocyte ratio (LMR). Comorbidities included diabetes, hypertension, and liver cirrhosis. Histopathological variables included tumor number, tumor diameter, tumor differentiation, vascular invasion, lymph node metastasis, and perineural invasion. The TNM stages were stratified according to the 8th edition of the AJCC Staging Manual. PMI was calculated as the total area of the psoas muscle in the horizontal axial imaging of the L3 vertebral body divided by the square of the body height (20–22). NLR, LMR, and PLR were calculated as the absolute counts of neutrophils, lymphocytes, and platelets, divided by the absolute counts of lymphocytes, monocytes, and lymphocytes, respectively. The cut-off value of CA19-9 was used at 37 U/mL (27).

### Acquisition of CT Radiomics Features

We followed the standardized procedures developed by the Imaging Biomarker Standardization Initiative to conduct the radiomics study (29). The study workflow is shown in **Figure 1**. Two experienced abdominal radiologists (YYJ and ZCY) reviewed transverse CT images to segment a region of interest (ROI) by MRIcroGL (www.mccauslandcenter.sc.edu) from arterial and venous phase CT images. Python software (version: 3.9.R) was used to conduct standardized processing on the two CT images, and then radiomics feature of arterial and venous phase CT images extraction were implemented.

R software (version 4.1.0) was used to analyze the data. The radiomics features of 40 patients were randomly selected for intra- and inter-observer correlation coefficient analysis. The intraclass correlation coefficient between the features extracted by the two ROIs drawn by YYJ was calculated. We calculated the inter-observer correlation coefficient between YYJ and ZCY and retained the intraclass correlation coefficient for both times >0.75 features. The features extracted for the first time from the ROI by YYJ were selected for subsequent analysis.

### Outcome

The primary outcome of our study was OS. OS was defined as the time interval between the day of surgery and the day of death for any reason. Recurrence-free survival (RFS) was defined from the date of surgery to the date of first ICC recurrence, death, or last follow-up visit. Postoperative follow-up strategies were as follows: once every 3 months in the first year after radical hepatectomy, once every 6 months up to 3 years after hepatectomy, and once a year in the following days. The last follow-up date for this cohort was April 2021.

### Development and Validation of Radiomics Model for Prediction of OS

Patients in the cohort were randomized into the training and trial groups (ratio: 7:3). The data from the training group were used to screen features and for radiomics model construction, and the testing group was used to validate it. We performed multiple feature selection to identify reliable and robust features while reducing redundancy. Firstly, the features with intraclass correlation coefficient >0.75 were selected by correlation analysis, and the features with high average absolute correlation were removed. Secondly, univariate Cox regression analyses were performed to identify OS-related radiomics features. Variables with *p* < 0.05 in the univariate Cox analyses were included into the Akaike information criterion (AIC) stepwise regression analysis, which took into account the statistical fit of the model and the number of parameters used for it. Before modeling, the global Schoenfeld test of each selected feature was utilized to perform the proportional hazards test. Then, the final hub radiomics features with the smallest AIC value were selected to establish the radiomics nomogram. Development and validation of the radiomics model accuracy was carried out by calibration, decision curve analysis (DCA), and area under the receiver operating characteristic curve (AUC).

### Development of the Clinical-Radiomics Model for Prediction of OS

The risk score of each patient was calculated based on the established radiomics model, and patients were divided into high- and low-risk groups according to the median risk score of the radiomics model. Univariate and multivariate Cox regression analyses were utilized to explore risk factors for OS. The global Schoenfeld test of each selected factor was utilized to perform the proportional hazards test, and a clinical-radiomics model was established to predict prognosis. The model was validated by calibration and AUC. More importantly, the bootstrap self-sampling method was repeated 1000 times as an internal validation method to calculate the reliable C-index. We compared the accuracy of prediction of ICC OS between the clinical-radiomics model and conventional staging system by C-index and AUC using CsChange package (30).

### Statistical Analyses

All statistical analyses were performed by SPSS (version 26.0) and R (version 4.1.0). Missing data were imputed using multiple imputations by logistic regression (**Supplementary Table 1**). Continuous variables between training and validation cohorts were compared by Student’s *t*-test or Mann–Whitney *U* test. Classification variables were compared by the x^2^ test. The optimal cut-off point of PMI was determined by the R survminer package. The cut-off values of PLR, NLR, and LMR for OS were calculated by X-tile software (31). We drew a survival curve through the Kaplan–Meier method with the log-rank test. Median OS was measured using the R survival package. OS-related variables were determined by univariate and multivariate Cox regression analyses. The python pyradiomics package was used to extract the radiomics features. The R forestplot package was used to visualize the Cox regression analysis results. The nomograms were established by the rms package. The C-index, AUC, DCA, and the calibration curves were applied to evaluate the performance of the nomogram. p (two sided) <0.05 was considered statistically significant.

## Results

### Clinicopathological Characteristics of Patients

We enrolled 199 ICC patients who underwent partial hepatectomy in our hospital between May 2011 and December 2020. After screening, 82 patients with mass-forming ICC constituted the study cohort. The cohort including 42 women and 40 men (**Table 1**). The median follow-up time was 42.1 months, and the 1-, 3-, and 5-year OS rates were 67.9%, 33.3%, and 19.4%, respectively. The 1-, 3- and 5-year RFS rates were 53.1%, 27.8%, and 16.7%, respectively. According to the optimal cut-off value of PMI, female patients with PMI ≤ 5.03 cm^2^/m^2^ and male patients with PMI ≤8.47 cm^2^/m^2^ were considered to have sarcopenia (**Supplementary Figures 1A, B**). The cut-off values of PLR, NLR, and LMR were 147.93, 2.53, and 2.92, respectively (**Supplementary Figures 2A–I**).

**Table 1:** Demographic and clinicopathological characteristics of ICC patients.

	All patients	Training group (n = 58)	Validation group (n = 24)	X^2^/T/Z	P
Age, years, average ± SD	63.49 ± 10.02	63.26 ± 9.50	64.04 ± 11.38	-0.320	0.750
Gender, n (%)				0.394	0.530
Male	40 (48.78%)	27 (46.55%)	13 (54.17%)		
Female	42 (51.22%)	31 (53.45%)	11 (45.83%)		
PMI, n (%)				0.236	0.627
High	41 (50.00%)	30 (51.72%)	11 (45.83%)		
Low	41 (50.00%)	28 (48.28%)	13 (54.17%)		
TNM stage, n (%)				1.829	0.401
III	22 (26.83%)	18 (31.03%)	4 (16.67%)		
II	17 (20.73%)	11 (18.97%)	6 (25.00%)		
I	43 (52.44%)	29 (50.00%)	14 (58.33%)		
Tumor differentiation, n (%)				2.061	0.151
High/middle	54 (65.85%)	41 (70.69%)	13 (54.17%)		
Low	28 (34.15%)	17 (29.31%)	11 (45.83%)		
Location, n (%)				3.397	0.065
Left	47 (57.32%)	37 (63.79%)	10 (41.67%)		
Right	35 (42.68%)	21 (36.21%)	14 (58.33%)		
Tumor size, cm, n (%)				0.394	0.530
>5	40 (48.78%)	27 (46.55%)	13 (54.17%)		
≤5	42 (51.22%)	31 (53.45%)	11 (45.83%)		
Tumor number, n (%)				0.000	1.000
Multiple	12 (14.63%)	8 (13.79%)	4 (16.67%)		
Single	70 (85.37%)	50 (86.21%)	20 (83.33%)		
Hepatitis B, n (%)				0.482	0.487
Positive	25 (30.49%)	19 (32.76%)	6 (25.00%)		
Negative	57 (69.51%)	39 (67.24%)	18 (75.00%)		
Lymph node invasion, n (%)				0.483	0.487
Yes	12 (14.63%)	10 (17.24%)	2 (8.33%)		
No	70 (85.37%)	48 (82.76%)	22 (91.67%)		
Vascular invasion, n (%)				4.823	0.028
Yes	13 (15.85%)	13 (22.41%)	0 (0.00%)		
No	69 (84.15%)	45 (77.59%)	24 (24.00%)		
Perineural invasion, n (%)				0.149	0.700
Yes	14 (17.07%)	11 (18.97%)	3 (12.50%)		
No	68 (82.93%)	47 (81.03%)	21 (87.50%)		
Cancer embolus, n (%)				4.196	0.041
Yes	19 (23.17%)	17 (29.31%)	2 (8.33%)		
No	63 (76.83%)	41 (70.69%)	22 (91.67%)		
Capsule invasion, n (%)				2.346	0.126
Yes	13 (15.85%)	12 (20.69%)	1 (4.17%)		
No	69 (84.15%)	46 (79.31%)	23 (95.83%)		
BMI, kg/m^2^, average ± SD				0.242	0.809
	22.38 ± 3.33	22.43 ± 3.48	22.24 ± 3.00		
Hepatolithiasis, n (%)				0.020	0.887
Yes	40 (48.78%)	28 (48.28%)	12 (50.00%)		
No	42 (51.22%)	30 (51.72%)	12 (50.00%)		
AFP, ng/ml, Median (IQR)				-0.025	0.980
	2.89 (2.54)	2.77 (1.83)	2.90 (2.55)		
CEA, μg/L, Median (IQR)				-1.035	0.301
	3.00 (3.33)	2.85 (3.63)	3.15 (3.03)		
CA199, U/ml, Median (IQR)				-0.357	0.721
	50.20 (542.53)	57.35 (434.98)	39.70 (1911.30)		
Diabetes, n (%)				1.238	0.266
Yes	16 (19.51%)	9 (15.52%)	7 (29.17%)		
No	66 (80.49%)	49 (84.48%)	17 (70.83%)		
Smoking, n (%)				0.129	0.719
Yes	21 (25.61%)	16 (27.59%)	5 (20.83%)		
No	61 (74.39%)	42 (72.41%)	19 (79.17%)		
Alcohol consumption, n (%)				0.099	0.754
Yes	17 (20.73%)	11 (18.97%)	6 (25.00%)		
No	65 (79.27%)	47 (81.03%)	18 (75.00%)		
Liver cirrhosis, n (%)				1.031	0.310
Yes	18 (21.95%)	11 (18.97%)	7 (29.17%)		
No	64 (78.05%)	47 (81.03%)	17 (70.83%)		
Albumin, g/L, average ± SD				-0.690	0.492
	38.87 ± 7.07	38.52 ± 9.67	39.71 ± 8.50		
PLR, n (%)				0.943	0.332
>147.93	41 (50%)	31 (53.45%)	10 (41.67%)		
≤147.93	41 (50%)	27 (46.55%)	14 (58.33%)		
NLR, n (%)				0.068	0.795
>2.53	53 (64.63%)	38 (65.52%)	15 (62.50%)		
≤2.53	29 (35.37%)	20 (34.48%)	9 (37.50%)		
LMR, n (%)				0.183	0.669
>2.92	38 (46.34%)	26 (44.83%)	12 (50%)		
≤2.92	44 (53.66%)	32 (55.17%)	12 (50%)		
ASA, n (%)				0.000	1.000
3-4	3 (3.66%)	2 (3.45%)	1 (4.17%)		
1-2	79 (96.34%)	56 (96.55%)	23 (95.83%)		

### Development and Validation of Radiomics Model in Mass Forming ICC Patients

Radiomics features from the arterial and venous phase images of ICC patients were extracted, and a total of 214 features were obtained from each patient, including shape features, first-order features, gray-level size zone matrix (GLSZM), gray-level run-length matrix (GLRLM), gray-level co-occurrence matrix (GLCM), neighboring gray-tone difference matrix (NGTDM), gray-level dependence matrix (GLDM).

**Figure 2:**
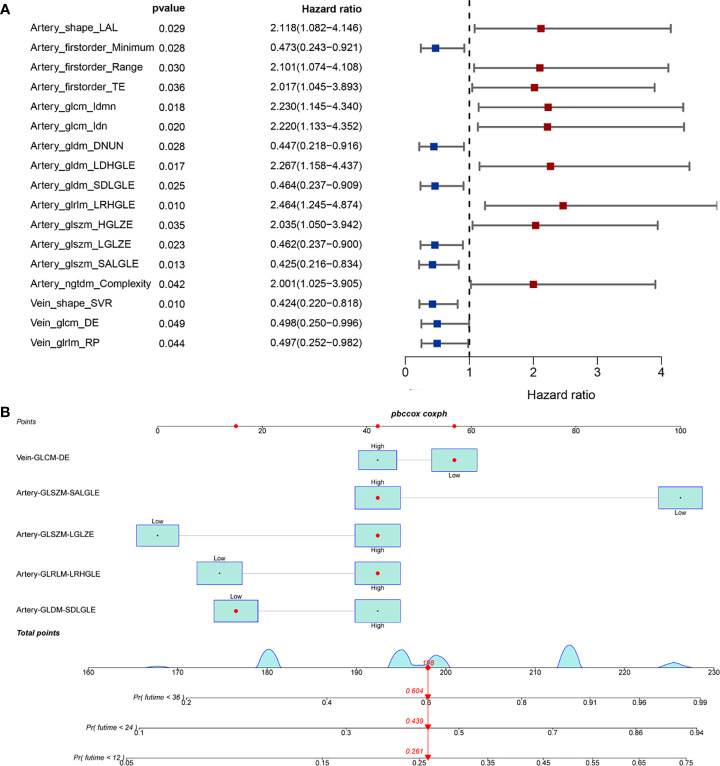
Development of radiomics model in mass forming ICC patients. **(A)** The univariable analysis identified the radiomics prognostic factors of OS; **(B)** Established radiomics nomogram of OS.

The training group comprised 58 patients, and the test group 24 patients. There were no significant differences in clinical characteristics between the training and validation cohorts, except for perineural and vascular invasion. The univariable Cox regression model was used to reduce the dimension of high-dimensional data, and 17 radiomics features of prognostic factors for OS were obtained (**Figure 2A**). AIC analysis showed that vein GLCM-DE, artery GLSZM-SALGLE, artery GLSZM-LGLZE, artery GLRLM-LRHGLE, and artery GLDM-SDLGLE were independent prognostic radiomics features for OS. We established a radiomics model that incorporated independent risk radiomics feature in OS (**Figure 2B**). As shown in **Supplementary Figure 3**, the P value of the global Schoenfeld test and features were all greater than 0.05, indicating that the model and each variable were satisfied with the proportional hazards test.

**Figure 3:**
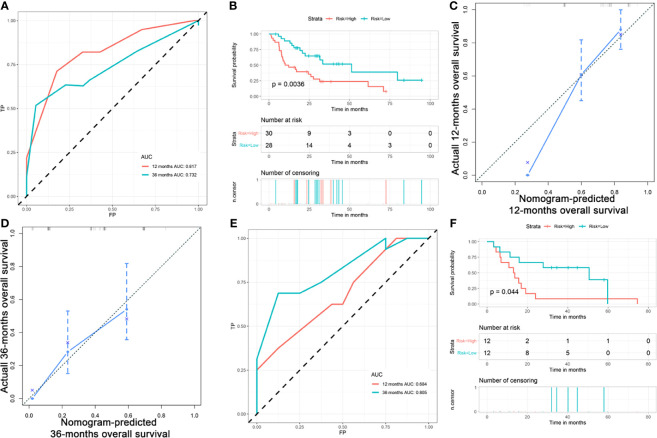
Validation of radiomics model in mass forming ICC patients. **(A)** AUC of radiomics model in the training cohort; **(B)** OS of patients with ICC in training cohort; **(C)** Calibration curve for predicting 1 -year survival in training cohort; **(D)** Calibration curve for predicting 3 -year survival in training cohort; **(E)** AUC of radiomics model in the validation cohort; **(F)** OS of patients with ICC in validation cohort by risk stratification.

In the training cohort, the radiomics model had an AUC of 0.817 (**Figure 3A**). The C-index was 0.692 [95% confidence interval (CI) 0.614-0.770]. We stratified ICC patients in the cohort by the model, and Kaplan-Meier survival curves showed that the median OS was 51.2 months for low-risk patients and 10.1 months for high-risk patients (*p*=0.0036) (**Figure 3B**). The predicted survival outcomes and actual observation showed good agreement by the calibration plot (**Figures 3C, D**). In the validation cohort, the AUC was 0.684 (**Figure 3E**) and the Kaplan-Meier survival analysis also showed good prognostic stratification (**Figure 3F**).

### Establishment of a Clinical-Radiomics Model for Predicting Prognosis in Mass-Forming ICC Patients

**Figure 4:**
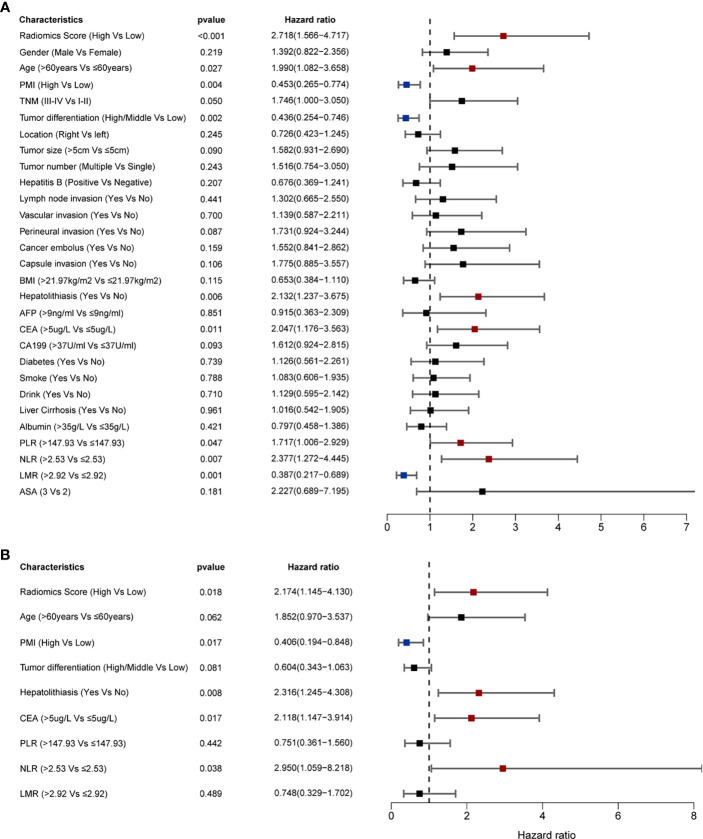
Prognostic factors of OS identified by univariable and multivariable Cox regression analyses. **(A)** Univariable analyses identified the factors of OS (*p* < 0.05). **(B)** Multivariable analyses identified the factors of OS (*p* < 0.05).

**Figure 5:**
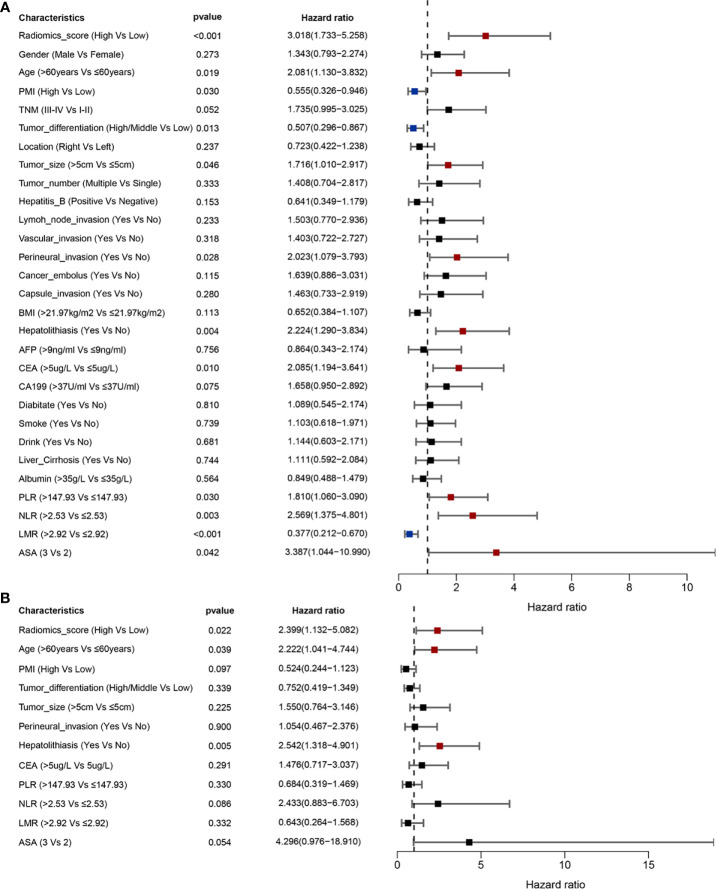
Prognostic factors of RFS identified by univariable and multivariable Cox regression analyses. **(A)** Univariable analyses identified the factors of RFS (*p* < 0.05). **(B)** Multivariable analyses identified the factors of RFS (*p* < 0.05).

**Figure 6:**
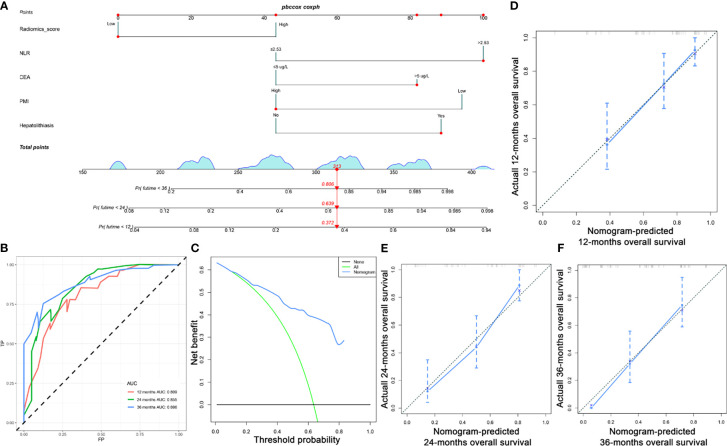
Establishment of ICC patients clinical radiomics model. **(A)** ICC survival nomogram **(B)** AUC of OS at 1, 2, and 3 years; **(C)** DCA of the model. **(D–F)** Calibration curve for predicting 1-, 2-, 3 -years survival.

**Figure 7:**
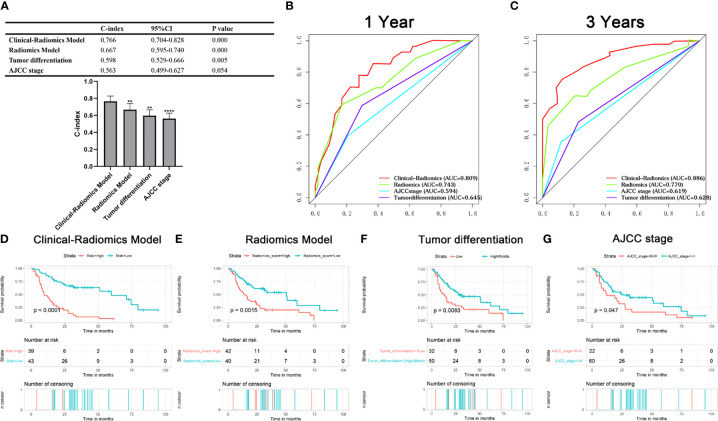
Kaplan-Meier survival curve, C-index, and AUC were compared among the models. **(A)** C-index of models; **(B, C)** AUC of the models for predicting OS at 1, and 3 years; **(D–G)** Kaplan-Meier survival curve showed OS risk stratification by the clinical-radiomics model, radiomics model, tumor differentiation systems and AJCC 8th edition for ICC patients.

The univariable analysis showed that PMI, radiomics score, age, hepatolithiasis, tumor differentiation, CEA, PLR, NLR, and LMR were risk factors for OS (**Figure 4A**). The multivariate analysis demonstrated that PMI, radiomics score, hepatolithiasis, CEA, and NLR were independent risk factors for OS (**Figure 4B**). The univariable analysis showed that radiomics score, age, PMI, tumor differentiation, tumor size, perineural invasion, hepatolithiasis, CEA, PLR, NLR, LMR, and ASA were risk factors for RFS (**Figure 5A**). The multivariate analysis demonstrated that PMI, radiomics score, age, and hepatolithiasis were independent risk factors for RFS (**Figure 5B**). The clinical-radiomics model for OS was constructed by PMI, radiomics score, hepatolithiasis, CEA, and NLR (**Figure 6A**). Similarly, as shown in **Supplementary Figure 4**, the P value of the global Schoenfeld test and factors were all greater than 0.05, indicating that the model and each variable were satisfied with the proportional hazards test. The reliable C-index (repeat 1000 times) of the clinical-radiomics model was 0.768 (95% CI, 0.765 to 0.770). The AUC of the clinical-radiomics model for predicting OS at 1, and 3 years was 0.809 and 0.886 (**Figure 6B**). DCA shows the prediction accuracy of the clinical-radiomics model in a wider range (**Figure 6C**). The predicted survival outcomes and actual observation has shown good agreement by the calibration plot (**Figures 6D–F**). We stratified ICC patients in the cohort by the clinical-radiomics model, and Kaplan-Meier survival curves showed that the median OS was 59.8 months for low-risk patients and 10.1 months for high-risk patients (**Figure 7D**) (*p<*0.0001).

### Comparison of Predictive Accuracy Between Clinical Radiomics Model and Other Models

We calculated the C-index to evaluate the consistency between predicted and actual values of all data and compared the C-index of clinical-radiomics model and other factors using Bootstrap self-sampling method. The C-index of the clinical-radiomics model was 0.766, which was significantly higher than the conventional staging systems (*p<*0.001) (**Figure 7A**). The C-indices of other models were 0.667 (radiomics model), 0.598 (tumor differentiation systems), 0.563 (AJCC 8^th^ edition). The AUC of the clinical-radiomics model for predicting OS at 1, and 3 years was 0.809 and 0.886, which was higher than for the radiomics model (0.743 and 0.770), tumor differentiation (0.645 and 0.628), and AJCC 8^th^ edition staging system (0.594 and 0.619) (**Figures 7B, C**). Clinical radiomics models showed better prognostic stratification in the cohort than the radiomics model, tumor differentiation system, and AJCC 8^th^ edition (**Figures 7D–G**).

## Discussion

In our cohort study, we established a clinical-radiomics model based on radiomics and sarcopenia to predict the prognosis of patients with mass-forming ICC who underwent curative resection. Our model consisted of five indicators, and stratified patients according to their risk score for OS. Compared with conventional staging systems, this model showed better performance, which indicated the utility of the model for predicting prognosis in ICC patients. This study is the first attempt to identify a comprehensive clinical-radiomics model combined with CT-derived radiomics features, sarcopenia, and clinical features that predict prognosis of patients with ICC undergoing radical hepatectomy, which is helpful for personalized therapeutic treatment.

Sarcopenia is an independent disease, but most clinicians do not know about the disease, let alone its role in cancer patients. A study reported that the incidence of sarcopenia was 1.6% in Europe (32), 3.4% in China (33), and 3.6% in the UK (34). Since its incidence is not low, we cannot ignore the influence of sarcopenia on tumor patients. Many studies have reported that sarcopenia has an influence on the prognosis of tumor patients (35, 36), and it is also an important factor in the prognosis of ICC patients (20–24). In our study, sarcopenia was also identified as an independent protective factor affecting the OS of ICC patients.

Radiomics is a new comprehensive discipline that combines artificial intelligence and medical imaging (37). It refers to the combination of quantitative features of images and clinical features of disease through high-throughput feature extraction to develop predictive models such as survival, and distant metastasis (38, 39). The goal is to help clinicians guide personalized precision treatment of patients (11, 40). Several studies have shown that radiomics can better predict the prognosis of mass forming ICC patients (41, 42). In our study, the integration of radiomics features from the arterial and venous phases provided more comprehensive features than from the arterial or venous phases alone. Our model also predicted patient outcomes well, which is consistent with previous studies.

Currently, there are many models for predicting tumor prognosis, including the AJCC staging system, radiomics model, and nomogram. Several studies have demonstrated that the nomogram is more precise than the traditional staging system in predicting tumor prognosis (43). It has been reported that the combined models have better predictive power than the individual models (13). Firstly, we established a prognostic nomogram of radiomics and verified the accuracy and reliability of prognostic prediction. Then, we integrated the radiomics model, clinicopathological features, and sarcopenia to develop a clinical-radiomics model to predict the outcome of ICC patients. The clinical-radiomics model included radiomics score, CEA, sarcopenia, hepatolithiasis, and NLR as independent prognostic factors. Compared with other models, the clinical-radiomics model has the following advantages. (1) Artificial intelligence is used to extract relevant imaging features, which can better reflect the real characteristics of tumors compared to conventional CT examination, which only provides tumor size. (2) The extracted radiomics features can only be used as a single indicator to reflect the imaging features of the tumor. In diagnosis or prognostic analysis, a single indicator cannot represent the overall situation of the patient. Our model incorporated radiomics features, clinical features, pathological features, special indicators, and routine indicators into the study. Compared with other models, our model can better reflect the overall situation of patients. According to our data, the 1-year AUC of the clinical-radiomics model was 0.809, which was higher than that of the radiomics model and AJCC staging model. (3) Radiomics can provide a convenient, non-invasive, low-cost, robust method to fully exploit the value of preoperative imaging. Compared with other models, the comprehensive model has improved the prediction, resulting in a significantly better performance in predicting outcomes than the radiomics model and conventional staging systems. Therefore, our clinical-radiomics model should play a role in personalized treatment.

Surgical excision is the only effective treatment for ICC. Preoperative identification of patients who can benefit from surgery and those who have poor postoperative prognosis is crucial in clinical practice. For patients with poor prognosis, it is possible to improve their prognosis by improving the prognostic indicators and giving some alternative therapies (44, 45). In our clinical-radiomics model, sarcopenia and NLR were indicators that could be ameliorated with clinical interventions. Many studies have reported that exercise and nutritional intervention can increase the quantity and quality of muscle in patients (46, 47), and it can improve the prognosis of patients with sarcopenia (48, 49). Therefore, based on the research findings and our models, improving sarcopenia in ICC patients may improve patient outcomes.

There were several limitations to this study. First, this was a small retrospective study, and potential selection bias cannot be excluded, and although we used standardized data processing to minimize these biases, there were still some deviations. Second, the diagnostic cut-off value for sarcopenia is still controversial, and we used the optimal OS-related cut-off value, which may have been biased. Third, the clinical-radiomics model only used the data from a single-center with limited patients (n=82), and it needs to be validated in prospective multicenter studies. Finally, it would be better to add the imaging features of the portal phase in this study.

## Conclusions

This study established a novel prognostic nomogram for predicting the prognosis of ICC after radical hepatectomy. The clinical-radiomics model integrating sarcopenia, clinical features, and radiomics score was the most accurate prognostic prediction for mass-forming ICC patients. It is provided an individualized prognostic evaluation in patients with mass-forming ICC and could help surgeons with clinical decision-making.

## Data Availability Statement

The original contributions presented in the study are included in the article/**Supplementary Material**. Further inquiries can be directed to the corresponding authors.

## Ethics Statement

All procedures of this retrospective cohort study and the ethical issues involved were reviewed and approved by the Institutional Review Board (IRB) of the First Affiliated Hospital of Wenzhou Medical University. The patients/participants provided their written informed consent to participate in this study. Written informed consent was obtained from the individual(s) for the publication of any potentially identifiable images or data included in this article.

## Author Contributions

LMD, YW, GC, ZPY conceptualized and designed the study. CYZ, HTY, JYZ, WMB, TD, CMZ, and LJW collected the data. CYZ and YJY provided computational analysis. LMD and BC statistical analyses and writing the manuscript. YW and GC review and revised the manuscript, administrative, and construct databases. All authors contributed to the article and approved the submitted version.

## Funding

This study was supported by National Natural Science Foundation of China (No. 82072685).

## Conflict of Interest

The authors declare that the research was conducted in the absence of any commercial or financial relationships that could be construed as a potential conflict of interest.

## Publisher’s Note

All claims expressed in this article are solely those of the authors and do not necessarily represent those of their affiliated organizations, or those of the publisher, the editors and the reviewers. Any product that may be evaluated in this article, or claim that may be made by its manufacturer, is not guaranteed or endorsed by the publisher.

## Supplementary Material

Supplementary Figure 1 The optimal cut-off value of male PMI was 8.47 **(A)**, and that of female PMI 5.03 **(B)**.

Supplementary Figure 2 The optimal cut-off value of PLR was 147.93 **(A–C)**, NLR was2.53 **(D–F)**, and LMR was 2.92 **(G–I)**.

Supplementary Figure 3 The global Schoenfeld test and individual Schoenfeld of five radiomics features in the training set.

Supplementary Figure 4 The global Schoenfeld test and individual Schoenfeld of five factors in the clinical-radiomics model.
