# Supplementary figures and images for: A Novel Clinical-Radiomics Model Based on Sarcopenia and Radiomics for Predicting the Prognosis of Intrahepatic Cholangiocarcinoma After Radical Hepatectomy
Source: Front Oncol. 2021 Nov 19;11:744311. doi: 10.3389/fonc.2021.744311 (PMC8639693; doi:10.3389/fonc.2021.744311)

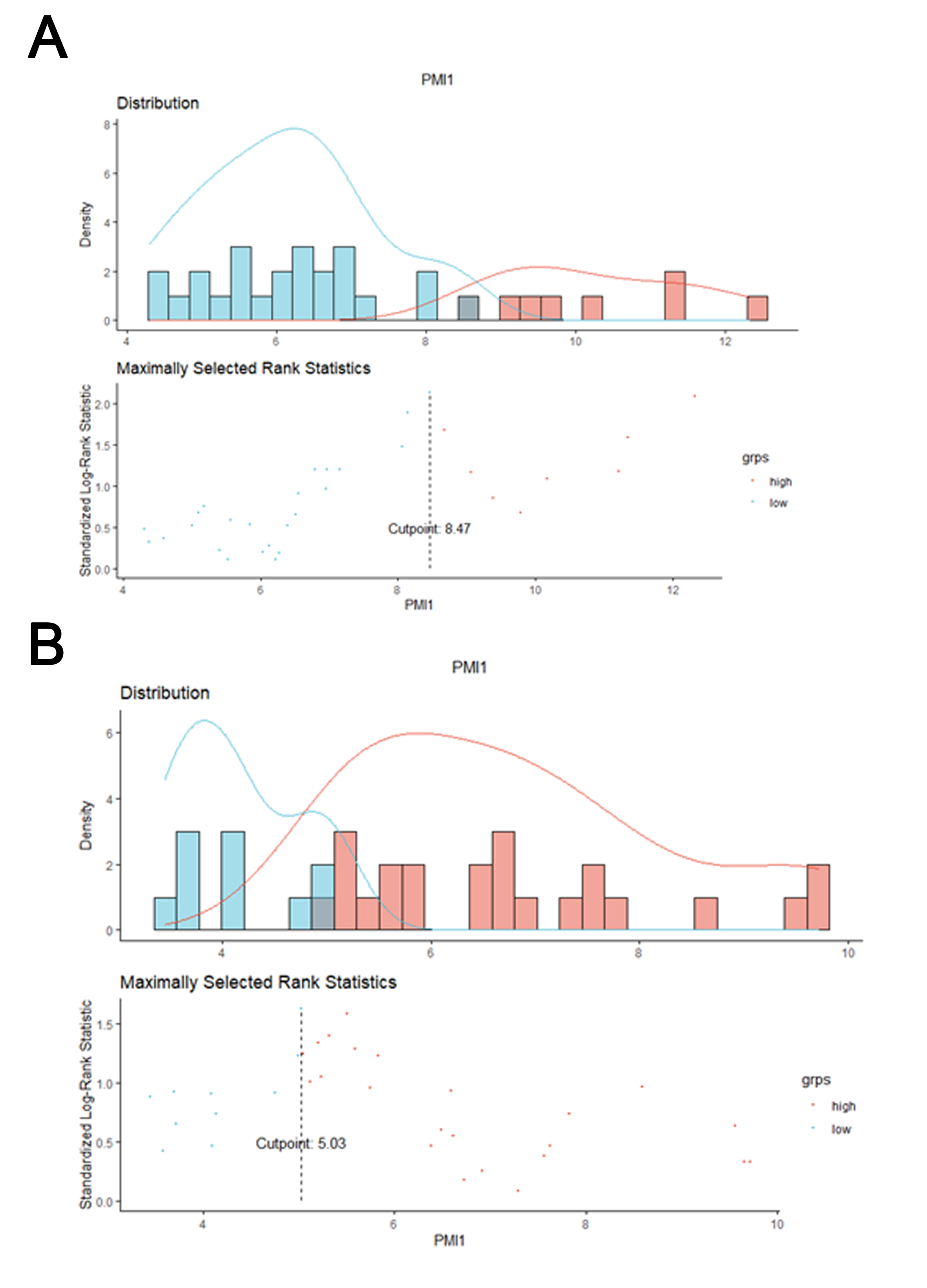

Supplement: Supplementary Figure 1 — The optimal cut-off value of male PMI was 8.47 (A), and that of female PMI 5.03 (B). [file Image_1.tif]

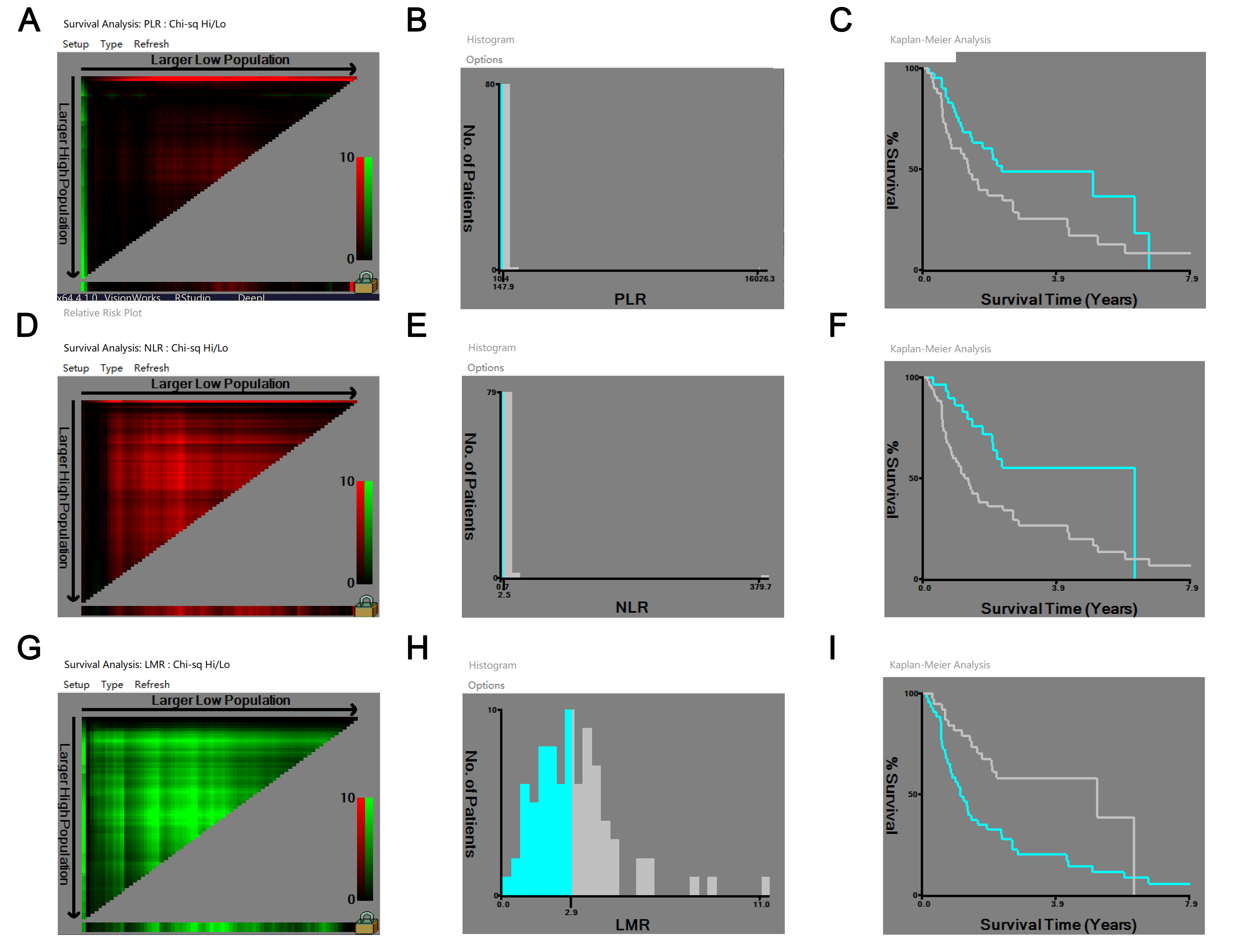

Supplement: Supplementary Figure 2 — The optimal cut-off value of PLR was 147.93 (A–C), NLR was2.53 (D–F), and LMR was 2.92 (G–I). [file Image_2.tif]

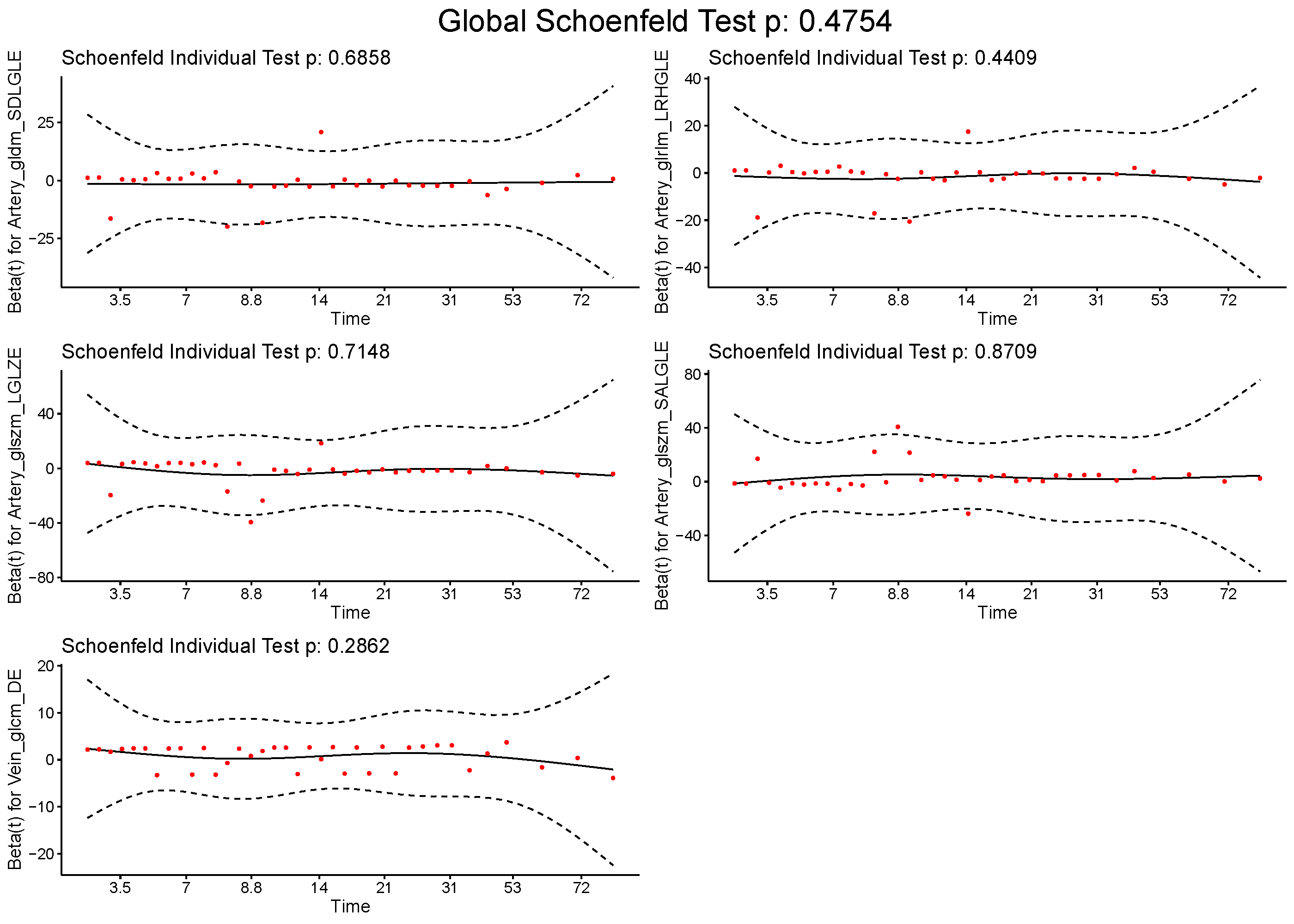

Supplement: Supplementary Figure 3 — The global Schoenfeld test and individual Schoenfeld of five radiomics features in the training set. [file Image_3.tiff]

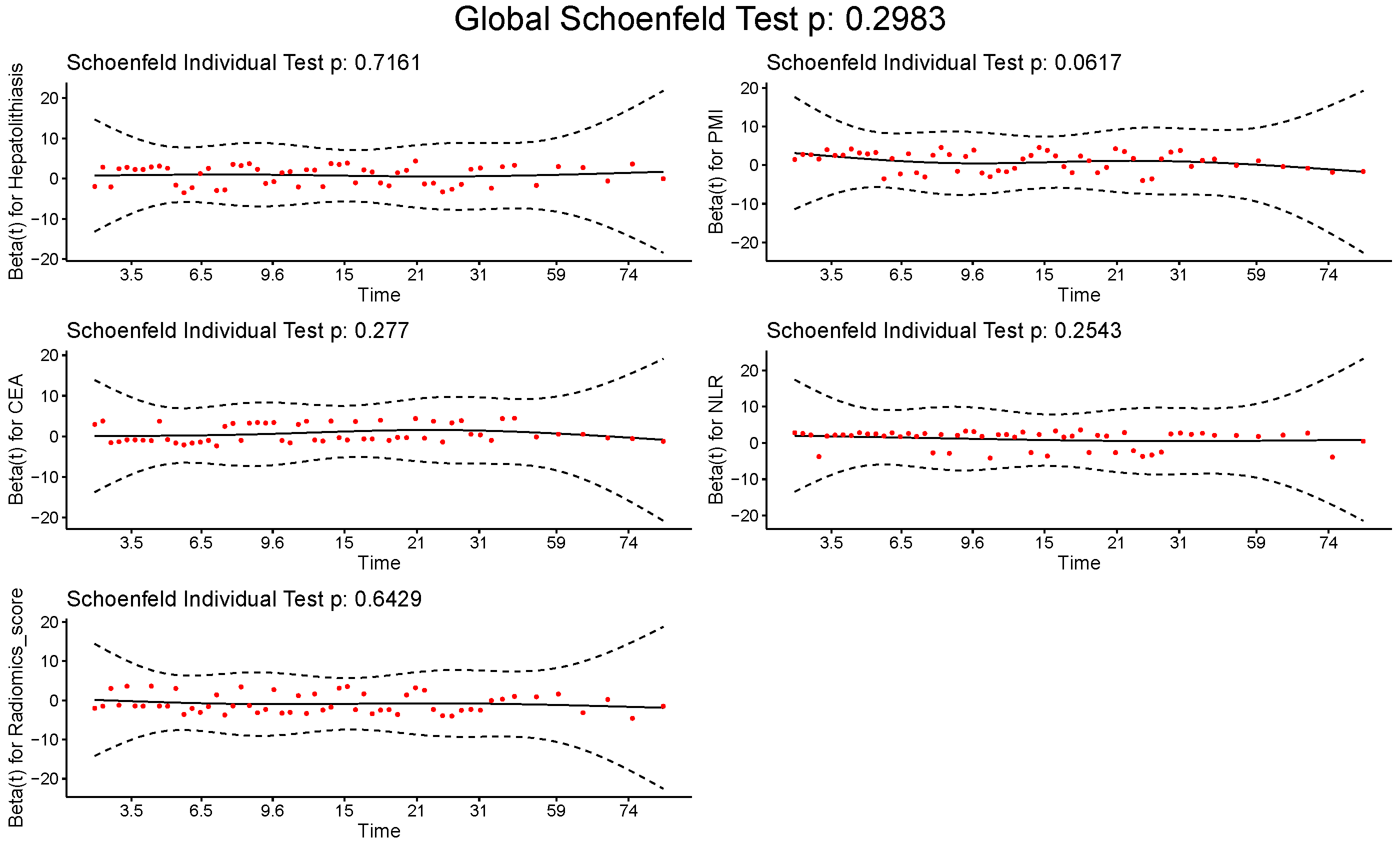

Supplement: Supplementary Figure 4 — The global Schoenfeld test and individual Schoenfeld of five factors in the clinical-radiomics model. [file Image_4.tiff]
