# Supplementary material for: A Novel Clinical-Radiomics Model Based on Sarcopenia and Radiomics for Predicting the Prognosis of Intrahepatic Cholangiocarcinoma After Radical Hepatectomy
Source: Front Oncol. 2021 Nov 19;11:744311. doi: 10.3389/fonc.2021.744311 (PMC8639693; doi:10.3389/fonc.2021.744311)
Supplement: Supplementary file 5 [file Table_1.docx]

**Table S1. Comparison of baseline characteristics between before and after imputation.**

| Characteristics | After imputation (n) | Before imputation (n) | Miss Value (n) | Training group (Miss Value, n)) | Validation group (Miss Value, n)) | Z | P |
| --- | --- | --- | --- | --- | --- | --- | --- |
| AFP, ng/ml, Median (IQR) | 2.89 (2.54) (82) | 2.84 (2.16) (80) | 2 | 2.74 (1.95) (1) | 2.90 (2.62) (1) | -0.287 | 0.774 |
| CEA, ug/L, Median (IQR) | 3.00 (3.33) (82) | 3.00 (3.40) (79) | 3 | 2.85 (3.68) (2) | 3.20 (3.10) (1) | -0.729 | 0.466 |
| CA199, U/ml, Median (IQR) | 50.20 (542.53) (82) | 59.5 (567.50) (79) | 3 | 61.75 (465.13) (2) | 45.20 (1964.60) (1) | -0.453 | 0.650 |
